# Effect of plant communities on bacterial and fungal communities in a Central European grassland

**DOI:** 10.1186/s40793-024-00583-4

**Published:** 2024-06-20

**Authors:** Clémentine Lepinay, Tomáš Větrovský, Milan Chytrý, Pavel Dřevojan, Karel Fajmon, Tomáš Cajthaml, Petr Kohout, Petr Baldrian

**Affiliations:** 1 Institute of Microbiology of the Czech Academy of Sciences, Vídeňská 1083, 14220 Prague 4, Czech Republic; 2 Department of Botany and Zoology, Faculty of Science, Masaryk University, Kotlářská 2, 61137 Brno, Czech Republic; 3 Regional Office Protected Landscape Area Bílé Karpaty, Nature Conservation Agency of the Czech Republic, Nádražní 318, 763 26 Luhačovice, Czech Republic

**Keywords:** Semi-natural grassland, Plant diversity, Fungal ITS, Bacterial 16S rRNA, Arbuscular mycorrhizal fungi

## Abstract

**Background:**

Grasslands provide fundamental ecosystem services that are supported by their plant diversity. However, the importance of plant taxonomic diversity for the diversity of other taxa in grasslands remains poorly understood. Here, we studied the associations between plant communities, soil chemistry and soil microbiome in a wooded meadow of Čertoryje (White Carpathians, Czech Republic), a European hotspot of plant species diversity.

**Results:**

High plant diversity was associated with treeless grassland areas with high primary productivity and high contents of soil nitrogen and organic carbon. In contrast, low plant diversity occurred in grasslands near solitary trees and forest edges. Fungal communities differed between low-diversity and high-diversity grasslands more strongly than bacterial communities, while the difference in arbuscular mycorrhizal fungi (AMF) depended on their location in soil versus plant roots. Compared to grasslands with low plant diversity, high-diversity plant communities had a higher diversity of fungi including soil AMF, a different fungal and soil AMF community composition and higher bacterial and soil AMF biomass. Root AMF composition differed only slightly between grasslands with low and high plant diversity. Trees dominated the belowground plant community in low-diversity grasslands, which influenced microbial diversity and composition.

**Conclusions:**

The determinants of microbiome abundance and composition in grasslands are complex. Soil chemistry mainly influenced bacterial communities, while plant community type mainly affected fungal (including AMF) communities. Further studies on the functional roles of microbial communities are needed to understand plant-soil-microbe interactions and their involvement in grassland ecosystem services.

**Supplementary Information:**

The online version contains supplementary material available at 10.1186/s40793-024-00583-4.

## Introduction

Grassland ecosystems cover a large portion of the earth’s surface and are present on almost all continents [88]. They play a fundamental role in providing many ecosystem services [103], and are a major carbon sink, storing more carbon than forest ecosystems [14, 26, 34]. Thus, the increasing atmospheric CO_2_ associated with global change will make the conservation and restoration of these ecosystems a major concern. Because of their central role in primary production, grasslands regulate numerous biogeochemical cycles. In addition to their carbon storage capacity, they store and transform several minerals in plant biomass and thus contribute to nutrient cycles that maintain soil fertility [45, 108]. Grasslands harbour a huge diversity of plant species, which is one of the main factors explaining their beneficial functions for the environment [9, 91, 103]. Consequently, grassland restoration is used to promote plant diversity and recover the resulting ecosystem services [83].

Plant diversity has been shown to ensure ecosystem stability in the face of global change by promoting ecosystem functions such as soil erosion reduction [8], plant community resistance to invasions [63], and the maintenance of insect diversity [83]. Plant diversity increases aboveground and belowground plant productivity [60], resulting in higher carbon storage [17]. In a field experiment, Tilman et al. [91] demonstrated that higher plant diversity allowed a more efficient use of limiting resources due to the diversity of plant metabolisms. High plant diversity is generally associated with relatively low levels of plant-available nutrients in soil because of their uptake in the plant biomass [55, 101]. In addition, plant diversity can influence the soil physicochemical components by increasing soil moisture and soil organic carbon content [101], or by modulating soil pH [32], although these effects were mainly found in experimental grasslands and were not universal. Thus, plant diversity levels can affect the soil microbial communities, either directly or through changes in soil characteristics. Lange et al. [55] found that the reduction of plant-available nutrients caused by high plant diversity could be compensated by stimulating the microbial component to obtain these nutrients. However, the functioning and interdependence of these plant-soil-microbe interactions remain largely unknown, especially in natural environments.

A meta-analysis of studies from several biomes showed that plant species diversity positively affects both bacterial and fungal biomass as well as microbial activity through an increase in microbial respiration [19]. Bennett et al. [7] demonstrated in an experimental grassland that soil with high plant diversity had a higher fungal-to-bacterial ratio and a higher biomass of arbuscular mycorrhizal fungi (AMF). A positive correlation between plant diversity and soil microbial diversity has also been frequently demonstrated in grasslands [41, 99, 106], but not consistently [70]. Upon closer inspection, Chen et al. [19] found that the biomass of some bacterial groups was negatively affected by plant diversity. The effect of plant diversity on bacterial diversity appears to depend on the bacterial phylum or bacterial functional guild [99]. Similarly, correlations between fungal diversity and plant diversity seem to depend on fungal functional guilds [106]. In a grassland, Wang et al. [99] found that AMF and saprotrophic fungi were the two guilds whose diversity was significantly positively correlated with plant diversity. The absence of correlation between plant species diversity and microbial community components, i.e. biomass, diversity and activity, has sometimes been explained by the soil chemical properties, which are affected by land use [36]. Indeed, previous studies found that the effects of plant diversity on fungal diversity were mediated by soil nutrients and texture [20, 89, 99]. Gastine et al. [36] also suggested considering plant functional diversity instead of plant species diversity to explain microbial diversity, but Eisenhauer et al. [31] demonstrated that plant species diversity, not only functional diversity, had a significant positive effect on microbial communities over a long time.

In natural conditions, gradients in plant species richness depend on environmental heterogeneity, degree of stress, disturbance and the intensity of competition [39, 46]. However, heterogeneous environments are not suitable for studying the effects of plant species richness on the diversity of soil microorganisms because it is difficult to decide whether the correlation between plants and microorganisms reflects the causal relationship between their diversities or an individual response of each of these groups to the variation in the environment. Therefore, we tried to reduce environmental heterogeneity by conducting our study in a wooded meadow with a homogeneous abiotic environment, i.e. deep soil on gentle slopes, where strong gradients in plant species richness were created by competition among plants, most notably by the asymmetric competition between trees and herbs. While treeless areas in this grassland are very rich in plant species [22, 104], grassland species richness decreases near forest edges, where herbaceous plants are affected by shading and lower soil moisture [86], and possibly also by root competition from trees. These differences in plant communities can affect both species richness of microbiomes and their species composition.

Here, we compare soil microbiomes between two contrasting types of grassland plant communities in a wooded meadow: species-poor, forest-edge grasslands versus species-rich grasslands in treeless patches. We analysed both the abundance and composition of bacterial communities, general fungal communities and AMF communities from soil, but also the composition of AMF communities in roots. We also characterized soil chemistry and plant community composition. Our aims were to explore (i) what are the effects of plant communities on soil microbiome composition and abundance, and (ii) what are the main drivers of bacterial, fungal and AMF community composition in such grasslands. We hypothesise that species-rich sites are associated with low soil nutrient content and high microbial biomass that helps obtain nutrients [55]. We expect that plant species richness increases the diversity of compounds released by plants into the soil [90], which favours the occurrence of microbial species that can utilize these compounds [100]. We also expect that both grassland types differ in microbiome composition because of the different composition of the plant-root community. As demonstrated in an experimental grassland, soil properties could be the main drivers of bacterial community composition [41], while plant community composition could be the main driver of fungal community composition, including AMF [41, 99].

## Materials and methods

### Study site

The studied semi-dry grasslands are located in the Čertoryje National Nature Reserve (48.8573° N, 17.4095° E) in the White Carpathians in the southeast of the Czech Republic. This grassland covers an area of about 7 km^2^ with an elevation of 320–584 m a.s.l. The climate is temperate subcontinental with a mean annual temperature of 8 °C and a mean annual precipitation of 700 mm. The soil is cambisol over flysch sediments [23]. The distinctive feature of the Čertoryje grassland is that it holds several world records for fine-scale vascular plant species richness [22, 104]. Factors favouring this high plant diversity include the long-term grassland continuity [42], its large area [65], and long-term continuous low-intensity management, mainly mowing and hay-making every June [54]. In addition, the large-scale homogeneity of soils across the White Carpathians combined with the fine-scale heterogeneity of habitat types support high plant species diversity [64, 65]. However, despite the high average plant species richness, the Čertoryje grassland is a mosaic of patches with high and low plant species richness. While species-rich plant communities occur in treeless patches, they become species-poorer at forest edges or near solitary trees [86].

### Selection of sampling plots

On 21st May 2019, sampling plots were selected based on a botanical survey that identified two grassland plant community types, one with low and one with high plant species diversity. Low-diversity plots were defined as containing no more than ten vascular plant species in 0.1 m^2^ (31.6 × 31.6 cm), while high-diversity plots contained at least 20 vascular plant species in an area of that size. Twelve plots of 0.1 m^2^ distributed in six pairs were sampled (Supplementary Table 1). Each pair consisted of one low-diversity plot close to a forest edge or a mature solitary oak tree, and one high-diversity plot in a treeless patch. The plots in each pair were located approximately 10 m from each other in similar abiotic conditions to limit environmental heterogeneity (Supplementary Fig. 1).

### Sample collection

All plant species visible above ground were recorded in each plot. Species were considered present in the plot if they were rooted there. Once the botanical survey was finished, living shoot biomass was collected in a plastic bag (litter and dead plant material were removed) in each plot. Then, a block of soil from the entire 0.1 m^2^ plot was collected to a depth of 10 cm using a spade and put on a clean plastic surface. The roots were carefully retrieved from the soil and stored in a plastic bag. Large roots (diameter > 2 mm), mainly from trees, were removed, as later milling of the whole piece would be too complicated. Stones and other debris particles were also removed. The soil was then sieved using a 5 mm mesh and homogenized in a large plastic bag. All materials and tools were washed with water and ethanol between sampling individual plots. Two 50 ml Falcon tubes were filled with soil for DNA extraction and PLFA/NLFA measurements and immediately frozen in liquid nitrogen, while two 50 ml Falcon tubes were filled with soil for soil chemistry measurements; all tubes were kept on dry ice. Material from all plots was processed on the same day as field sampling, and the two plots in a pair were processed simultaneously. In the laboratory, the shoots and roots were stored at 4 °C and the soil samples at − 80 °C.

### Soil chemistry

Soil chemical analyses were performed in a laboratory of the Institute of Botany (Průhonice, Czech Republic). Active and exchangeable pH were determined after mixing 5 ml of soil in 25 ml of H_2_O and KCl 0.1 M, respectively (ISO 10390:2005(E)). Total N and C contents were determined using a FLASH 2000 Elemental Analyzer (Thermo Scientific). Organic C content was determined after decomposition in HCl [71]. Total Ca, Mg and K contents, expressed in mg kg^−1^, were measured after extraction in ammonium acetate pH 7. Ca and Mg contents were eluted in sulfuric acid and lanthanum chloride, respectively, while K was determined from the original extract. These nutrient contents were measured using a high-resolution continuum source atomic absorption spectrometer contrAA 700 (Analytik Jena AG). Exchangeable P content, in mg kg^−1^, was determined by the Olsen’s method [76]. Nitrate and ammonium ions, expressed in mg kg^−1^, were measured by spectrophotometry coupled with flow injection analysis (QuikChem 8500 Series 2, Hach company). Finally, cation exchange capacity (CEC) was measured in mmol kg^−1^ after extraction in 0.1 M barium chloride solution with a high-resolution continuum source atomic absorption spectrometer contrAA 700 (Analytik Jena AG). Soil moisture, expressed in %, was calculated as the difference in mass before and after drying to constant mass at 105 °C (ISO 11465:1993).

### Quantification of microbial biomass

Bacterial and fungal biomasses were estimated with a phospholipid fatty acid (PLFA) analysis, while AMF biomass was estimated with a neutral lipid fatty acid (NLFA) analysis. All the analyses were performed by the Laboratory of Environmental Biotechnology (Institute of Microbiology, Prague, Czech Republic) as described in Frouz et al. [33]. Briefly, total lipids were extracted from 1 g of freeze-dried soil sample with a mixture of chloroform:methanol:phosphate (1:2:0.8) and separated using solid-phase extraction cartridges (LiChrolut Si 60, Merck). The obtained neutral lipids and phospholipids fractions were eluted in 2 mL of chloroform and 2 mL of methanol, respectively, and subjected to mild alkaline methanolysis. The free methyl esters of NLFA and PLFA were analysed by gas chromatography (Varian 3400 GC) coupled with mass spectrometry (Finnigan ITS-40). Fungal biomass was estimated based on the 18:2ω6,9 content. Bacterial biomass was estimated as the sum of i14:0, i15:0, a15:0, 15:0, i16:0, 16:1ω9, 16:1ω7, 16:1ω5, 10Me-16:0, i17:0, a17:0, cy17:0, 17:00, 10Me-17:0, 18:1ω7, 10Me-18:0 and cy19:0. AMF biomass was estimated based on the 16:1ω5 content of the neutral lipids fraction. Total microbial biomass was estimated as the sum of all PLFA.

### Shoot and root processing

Shoots were weighed for fresh biomass, frozen at − 40 °C and lyophilized to obtain dry shoot biomass and shoot water content. Roots were cleaned with water, dried with paper towels and weighed to determine fresh biomass. Then, roots were cut into 1 cm pieces, frozen at − 40 °C and lyophilized to determine root water content. The total amount of roots sampled for each plot was milled in a fine powder in an Ultra Centrifugal Mill ZM 200 (Retsch). One 50 ml Falcon tube of finely milled roots was randomly subsampled for each plot, of which three 50 mg replicates were collected and stored at − 40 °C until freeze-drying for DNA extraction.

### Root DNA extraction, amplification of plant and AMF communities and sequencing

Root DNA was extracted and purified using the chloroform-based protocol of Miller et al. [66] with Sagova-Mareckova’s modifications [84]. The three purified root DNA extracts obtained were pooled, and from this pool, the PCRs for amplification of plant and AMF communities were done in triplicate. To cover a wide range of plant diversity, the internal transcribed spacer 2 (ITS2) region of the nuclear ribosomal DNA (rDNA) was amplified with the primers ITS2-S2F (5′-ATGCGATACTTGGTGTGAAT-3′; [15]) and ITS4 (5′-TCCTCCGCTTATTGATATGC-3′; [102]) to characterize the plant community composition. For the characterisation of the root AMF community, a part of the small subunit (SSU) of the rDNA was amplified with the primers NS31 (5′-TTGGAGGGCAAGTCTGGTGCC-3′) and AML2 (5′-GAACCCAAACACTTTGGTTTCC-3′) [69]. The PCRs were carried out with 1 μl of DNA (at 5 ng/μl) in a 25 µl reaction mixture containing 5 μl of Q5 Reaction Buffer (New England Biolabs, Inc.), 0.5 μl of 10 mM PCR Nucleotide Mix (Bioline), 1.5 μl of bovine serum albumin at 10 mg ml^−1^ (GeneON), 0.25 μl of Q5 High-Fidelity DNA polymerase (New England Biolabs, Inc.), 5 μl of Q5 High GC Enhancer (New England Biolabs, Inc.), 1 μl of 10 µM of each primer (Sigma-Aldrich) and 9.75 μl of H_2_O. For the plant community, the PCR amplification conditions were 94 °C for 5 min, 35 cycles of 30 s at 94 °C (denaturation), 56 °C for 30 s (annealing) and 72 °C for 45 s (extension), followed by 10 min at 72 °C (final extension). For AMF root community, the PCR amplification conditions were 98 °C for 30 s, 32 cycles of 10 s at 98 °C, 30 s at 63 °C and 20 s at 72 °C, followed by 2 min at 72 °C. The triplicate PCR products were then pooled, purified using a MinElute kit (Qiagen) and quantified with a Qubit™ dsDNA BR Assay kit (Thermo Fisher Scientific) on a Qubit 2.0 fluorometer (Life Technologies). An amplicon library was prepared from the purified PCR products using the TruSeq DNA PCR-free kit (Illumina), and sequenced in-house with a MiSeq Reagent Kits v2 (Illumina) on an Illumina MiSeq platform (2 × 250 base paired-end reads).

### Soil DNA extraction and metabarcoding of microbial communities

Soil DNA was extracted from 1 g of frozen soil using the RNeasy PowerSoil Total RNA kit (Qiagen), followed by the RNeasy PowerSoil DNA Elution Kit (Qiagen). Three extractions were performed for each sample. The three soil DNA extracts were then pooled. Three PCR reactions were performed for each sample and each primer set. For the characterisation of bacterial, fungal and AMF soil communities, the V4 region of bacterial 16S rRNA gene, the ITS2 region of fungal rRNA gene and a part of the SSU of AMF rRNA gene were amplified. The primer sets used for bacteria, fungi and AMF were 515F/806R [13], gITS7/ITS4 [47] and NS31/AML2 [69], respectively. Apart from the primers used, the PCR reaction mixtures were the same as the mixture used for creating the root amplicon libraries. The PCR amplification conditions for bacteria were 94 °C for 4 min, 25 cycles of 45 s at 94 °C, 50 °C for 1 min and 72 °C for 75 s, followed by 10 min at 72 °C, and for fungi 94 °C for 5 min, 30 cycles of 30 s at 94 °C, 56 °C for 30 s and 72 °C for 30 s, followed by 7 min at 72 °C. For the soil AMF community, the PCR amplification conditions were the same as previously described for the root AMF community. The three PCR products were pooled, purified, quantified and sequenced as previously described for the root amplicon libraries.

### Bioinformatic processing

All amplicon sequencing data were analysed with the SEED2 pipeline [95].

For plant ITS2 sequencing data, paired-end reads were joined using fastq-join [2]. Then, quality filtering was performed to remove sequences with a minimum average sequence quality < 30. All plant ITS2 fragments, regardless of length, were extracted using the ITSx software [6]. Among the ITS2 fragments obtained, sequences < 150 bases were removed. Chimeric sequences were then identified and removed using USEARCH 8.1.1861 [29], and the remaining sequences were clustered into operational taxonomic units (OTUs) using UPARSE implemented in USEARCH [30] with a 98% similarity level. The global singletons, i.e. OTUs represented by only one sequence across all the samples, were ignored for the rest of the analyses. The most abundant sequence from each OTU cluster was chosen as the representative sequence for taxonomic identification of that cluster. The closest hits at the species level were identified using the Basic Local Alignment Search Tool (BLASTn) of the NCBI against a local database containing the representative ITS2 sequences of the plant species collected in the same grassland. A second identification was carried out using BLASTn against the Genbank database. The OTUs with no hits in both BLASTn searches were removed. The results of the first BLASTn search were used when OTUs had a similarity > 98%. For the OTUs having a similarity ≤ 98% in the first BLASTn search, the results of the second BLASTn search were used. The plant genera were used to assign putative mycorrhizal type using the database created by Soudzilovskaia et al. [87].

For AMF sequencing data from root and soil compartments, we used only the forward reads for the analyses. Quality filtering was performed to remove the sequences with minimal average sequence quality < 30 and the sequences < 70 bases. Then, as described above, chimeric sequences were identified and removed, and the remaining sequences were clustered into OTUs with a 97% similarity level. The global singletons were ignored for the rest of analyses. The representative sequence from each OTU cluster was selected as described above. The closest hits at the genus level were identified using BLASTn against the MaarjAM database [77] containing 20 399 AMF 18S sequences distributed in 352 virtual taxa (status October 2016). The OTUs were assigned to virtual taxa from MaarjAM database if they had an *E*-value < 10^−50^, a coverage ≥ 90% and a similarity ≥ 97% [94]. Among the rest of the OTUs not fitting with these defined thresholds, a second identification was carried out using BLASTn against the Genbank database. The OTUs with non-fungal hits, no hits, or where the class is different from Glomeromycetes, in both BLASTn searches, were removed. For the OTUs with no hits in one of the BLASTn search but identified as Glomeromycetes in the other BLASTn search, we kept the BLASTn search with identification.

For fungal ITS2 and bacterial 16S rRNA gene sequencing data, paired-end reads were joined as described above. Then, a quality filtering was performed to remove the sequences with minimal average sequence quality < 30. For fungi, the ITS2 region, even if incomplete, was extracted using the ITSx software [6] before processing. Among the ITS2 fragments obtained, sequences < 40 bases were removed. Among the bacterial 16S rRNA gene sequences, those < 200 bases or > 350 bases were removed. Then, for both fungi and bacteria, the chimera removal, clustering at 97% similarity level, removal of global singletons and selection of the representative sequence from each OTU cluster, were performed as described above. The identification of fungal and bacterial sequences was performed using BLASTn against the UNITE 9.0 database [73] and the Ribosomal Database Project [25], respectively. The identification was available at species level for fungi and genus level for bacteria. The non-fungal and non-bacterial hits and sequences without identification were removed. For fungi, the OTUs having the same species identification, a similarity ≥ 97% and a coverage ≥ 95% were merged into a single taxon, and species-level identification was used. For the OTUs with lower similarity, lower coverage or both, genus-level identification, or the best available identification, was used. The fungal genera were used to assign putative ecophysiological guilds using the FungalTraits database [80].

The use of molecular taxa (here OTUs) has some limitations for the characterization of environmental microbiomes due to the fact that their identity as biological species, especially for those taxa that are not yet known to science, is not clear. It should be noted that the numbers of observed sequences of each taxon should not be considered as precise estimates of relative taxon abundances. Despite these limitations, the use of OTUs (i.e. molecular species) is the most widely used approach in the analysis of fungal and other microbiomes [50].

The raw sequence data for the communities of plants, soil bacteria, soil fungi, soil AMF and root AMF have been deposited into the National Center for Biotechnology Information database under the accession number PRJNA891626.

### Statistical analyses

Statistical analyses were performed using R version 4.1.2 (R Core Team, R Foundation for Statistical Computing, Vienna, Austria). For the analyses, plant richness was calculated as the total number of genera per plot, considering the genera identified in the botanical survey of aboveground vegetation and adding the missing genera found by the sequencing analysis of plant roots.

Soil chemical variables, microbial variables (i.e. PLFAs, NLFA and diversity indices), and plant community variables (i.e. plant richness, dry shoot biomass, shoot and root water content and distance to nearest tree) were compared between high and low-diversity plots using Student t-tests after each variable was tested for normality and variance homogeneity using the Shapiro–Wilk and Bartlett tests, respectively. Diversity indices selected for characterizing microbial communities were those commonly used in microbial ecology, i.e. Shannon, evenness, Chao1 and ACE [51].

For the diversity analyses of plant and microbial communities, the number of sequences for each sample was randomly subsampled at the same sequencing depth (Supplementary Fig. 2). For the plant community, each sample was subsampled at 9302 sequences. For the root and soil AMF communities, each sample was subsampled at 2247 sequences, but three samples in the root AMF community were below this threshold and were not considered for diversity analyses. For fungal and bacterial communities, each sample was subsampled at 10 349 and 8400 sequences, respectively. Diversity indices of each community were calculated using the ‘vegan’ R package [74].

For the analyses of community composition and similarity, the same datasets were used, but for the root AMF community, the three samples with sequencing depth below the defined threshold were included with all their sequences. To determine the significance of diversity level for the plant and microbial communities, a permutational multivariate analysis of variance (PERMANOVA) was used. Plant and microbial community composition were visualized with a two-dimensional non-metric multidimensional scaling (NMDS) ordination analysis based on the Bray–Curtis distances of the relative abundances of OTUs, using the ‘metaMDS’ function from the ‘vegan’ R package. Then, all the variables (i.e. soil chemicals, PLFAs and NLFA and plant community variables) were fitted as vectors in the NMDS analyses using the ‘envfit’ function from the ‘vegan’ R package to identify the variables significantly correlated with the community composition. To avoid multicollinearity among the variables significantly correlated with the community composition, the variance inflation factor (VIF) was calculated for each variable using the ‘vif.cca’ function of the ‘vegan’ R package, and the variables with a VIF value > 10 were removed. The importance of the remaining variables in explaining variance in community composition was determined using variation partitioning analyses on Hellinger-transformed OTU abundances. The ‘varpart’ function from the ‘vegan’ R package was used. The significance of the obtained variances was tested with redundancy analyses performed with the ‘rda’ function from the ‘vegan’ R package.

## Results

### Associations between plant community, soil chemistry and microbial biomass

All the plant community variables were significantly associated with the plant diversity level, being higher in the high-diversity plots than in the low-diversity plots (Table 1).

**Table 1:** Plant, soil chemical and microbial characteristics in low and high-diversity plots from the Čertoryje grassland

	Low diversity	High diversity	Student’s t-test	*P* value
Vegetation variables				
Plant richness (number of genera)	9.2 ± 1.0	24.7 ± 2.0	7.28	** < 0.001**
Dry shoot biomass (g)	8.5 ± 1.8	18.4 ± 2.3	3.38	**0.008**
Distance to nearest tree (m)	2.0 ± 0.5	4.9 ± 0.8	3.16	**0.014**
Shoot water content (%)	67.8 ± 1.5	72.5 ± 0.9	2.74	**0.025**
Root water content (%)	67.6 ± 1.1	71.5 ± 1.2	2.48	**0.033**
Chemistry variables				
pH (H_2_O)	5.94 ± 0.15	6.51 ± 0.38	1.39	0.208
pH (KCl)	5.40 ± 0.14	6.01 ± 0.39	1.47	0.188
Total N (%)	0.34 ± 0.02	0.47 ± 0.05	2.61	**0.029**
Total C (%)	4.49 ± 0.31	5.89 ± 0.54	2.41	**0.038**
C/N	13.22 ± 0.42	12.65 ± 0.32	− 1.1	0.317
Organic C (%)	3.71 ± 0.27	4.72 ± 0.29	2.58	**0.028**
Inorganic C (%)	0.78 ± 0.09	1.17 ± 0.3	1.24	0.252
Ca (mg/kg)	3844 ± 323	5899 ± 1283	1.54	0.169
Mg (mg/kg)	440 ± 30	407 ± 28	− 0.8	0.456
K (mg/kg)	296 ± 42	314 ± 31	0.43	0.679
P (mg/kg)	3.83 ± 0.62	4.77 ± 0.53	1.22	0.260
NH_4_^+^ (mg/kg)	8.40 ± 1.84	15.33 ± 5.14	1.66	0.131
N–NH_4_^+^ (mg/kg)	6.52 ± 1.43	11.90 ± 3.99	1.66	0.131
NO_3_^−^ (mg/kg)	14.36 ± 0.95	11.98 ± 0.61	− 2	0.074
N–NO_3_^−^ (mg/kg)	3.25 ± 0.21	2.71 ± 0.14	− 2	0.075
CEC (mmol/kg)	265 ± 15	320 ± 43	1.14	0.293
Soil water content (%)	25.54 ± 1.03	24.05 ± 2.87	− 0.7	0.531
Microbes variables				
Fungal biomass (PLFA fungi, ppm)	1.24 ± 0.40	1.56 ± 0.17	0.73	0.492
Bacterial biomass (PLFA bacteria, ppm)	12.60 ± 1.90	19.47 ± 1.62	2.75	**0.021**
F/B ratio (PLFA)	0.09 ± 0.02	0.08 ± 0.01	− 0.3	0.803
Total microbial biomass (total PLFA, ppm)	18.95 ± 2.95	28.62 ± 2.21	2.62	**0.027**
AMF biomass (NLFA, ppm)	3.63 ± 0.97	20.1 ± 2.92	6.72	** < 0.001**
AMF/Fungi ratio (NLFA/PLFA fungi)	3.25 ± 0.41	13.39 ± 2.23	7.78	** < 0.001**
AMF/total ratio (NLFA/total PLFA)	0.19 ± 0.03	0.73 ± 0.13	6.34	** < 0.001**

Values are means ± standard errors. Comparisons of values between low and high-diversity plots are performed using Studentʼs t-tests and associated *P* values. Significant differences between low and high-diversity plots are indicated by a bold *P* value (*P* < 0.05)

**Fig. 1:**
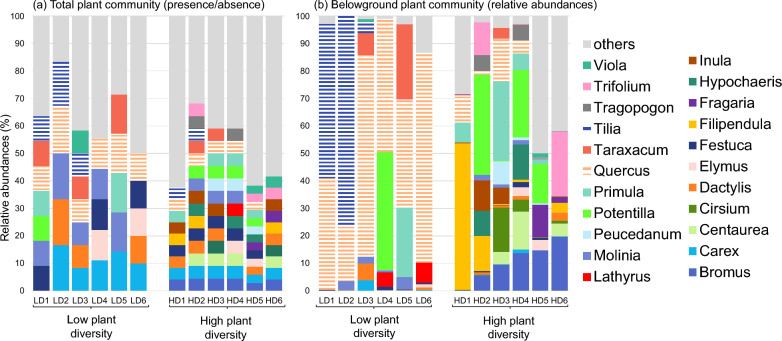
Plant composition in species-poor and species-rich grasslands. Plant genera **a** from the botanical survey completed by the ITS sequencing analysis, represented as a presence-absence matrix, and **b** from the OTU abundance matrix resulting from the ITS sequencing analysis. Only plant genera with relative abundance of > 1% in more than one of the 12 plots are represented. The tree genera mentioned in the text, i.e. *Quercus* and *Tilia*, are highlighted by horizontal stripes

A striking difference between the botanical survey, documenting the aboveground part of the plant communities, and the root sequencing data was the predominance of trees (*Quercus* and *Tilia*) in the belowground part of low-diversity plots (Fig. 1, Supplementary Table 2). On average, *Quercus* and *Tilia* accounted for 50% and 23% of relative abundances, respectively, among all the genera found in the low-diversity plots, compared to only 5% and 0.03% in the high-diversity plots, respectively.

Potential mycorrhizal types based on plant genera identified by root sequencing analysis showed a dominance of ectomycorrhizal plants in low-diversity plots and a dominance of arbuscular mycorrhizal plants in high-diversity plots (Supplementary Fig. 3).

Few soil chemical variables differed between the two types of plant communities (Table 1). Only total N, total C and organic C contents were significantly higher in the high-diversity plots than in the low-diversity plots, while there were no significant differences in the other soil chemical variables.

Looking at the biomarkers of microbial biomass, arbuscular mycorrhizal fungi (AMF) were most different between the two plant community types (Table 1). AMF biomass (NLFA), AMF biomass to fungal biomass ratio (NLFA/PLFA fungi) and AMF biomass to total microbial biomass ratio (NLFA/total PLFA) were significantly higher in the high-diversity plots than in the low-diversity plots. The same trends were observed for bacterial biomass (PLFA bacteria) and total microbial biomass (total PLFA).

### Effects of plant community on bacterial community diversity and composition

**Fig. 2:**
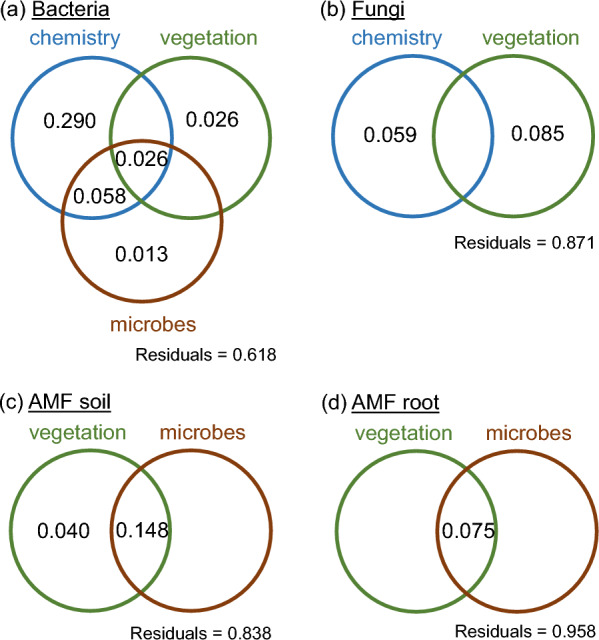
Venn diagrams visualizing the variance partitioning of **a** bacterial communities, **b** general fungal communities, **c** AMF communities from soil, and **d** AMF communities from roots into chemical variables, plant community type variables and microbial variables. Numbers are adjusted R^2^ values. Values < 0 are not shown

The two types of plant communities did not differ in bacterial community composition (Table 2a, Supplementary Fig. 4a) or bacterial community diversity (Supplementary Table 3). All the categories of variable used, i.e. plant community, chemistry and microbial variables, were significantly correlated with bacterial community composition. CEC and pH(H_2_O) were the most important determinants of bacterial community structure (Table 2b). Variation partitioning showed that the observed variance in bacterial community composition was significantly explained by the chemical variables only (Table 2c, Fig. 2a). Both the pure effect of chemistry and the joint effect of chemistry, plant community and microbes were significant. The residual variation, i.e. the variation not explained by any of our variables, was 62%.

**Table 2:** Factors influencing microbial community composition

	Bacteria					Fungi					AMF soil					AMF root				
	Df	Sum of squares	R2	F	*P* value	Df	Sum of squares	R2	F	*P* value	Df	Sum of squares	R2	F	*P* value	Df	Sum of squares	R2	F	*P* value
(a) Adonis F-statistics from PERMANOVA tests																				
Diversity (low/high)	1	0.145	0.14	1.56	0.184	1	0.65	0.16	1.87	**0.006**	1	0.86	0.38	6.13	**0.002**	1	0.52	0.18	2.23	**0.046**
Residual	10	0.932	0.87			10	3.47	0.84			10	1.4	0.62			10	2.34	0.82		
Total	11	1.077	1			11	4.12	1			11	2.26	1			11	2.87	1		

	r2	*P*				r2	*P*				r2	*P*				r2	*P*			
(b) Environmental variables significantly correlated with the community composition from NMDS ordinations																				
Vegetation variables																				
Dry shootbiomass	–	–				0.88	0.001				0.65	0.008				–	–			
Plant richness	0.59	0.014				0.70	0.008				0.64	0.01				0.54	0.029			
Chemistry variables																				
pH (H_2_O)	0.87	0.004				0.87	0.001				–	–				–	–			
Organic C	–	–				0.64	0.009				–	–				–	–			
Inorganic C	0.65	0.007				0.66	0.008				–	–				–	–			
CEC	0.92	0.001				0.92	0.001				–	–				–	–			
Total C	0.73	0.004				–	–				–	–				–	–			
Microbes variables																				
AMF biomass	0.79	0.001				–	–				–	–				–	–			
AMF/fungi ratio	–	–				–	–				0.55	0.039				–	–			
Totalmicrobial biomass	–	–				–	–				–	–				0.65	0.008			

	Df	Adjusted R2	F	*P* value		Df	Adjusted R2	F	*P* value		Df	Adjusted R2	F	*P* value		Df	Adjusted R2	F	*P* value	
(c) Variation partitioning analysis using environmental variables significantly correlated with NMDS ordinations																				
Vegetation variables																				
Pure effect	1	0.026	1.25	0.288		2	0.085	1.34	0.152		2	0.040	1.24	0.222		1	− 0.010	0.89	0.567	
Joint effect	1	0.022	1.24	0.228		2	0.070	1.41	**0.003**		2	0.188	2.27	**0.014**		1	0.064	1.76	0.076	
Chemistry variables																				
Pure effect	4	0.290	2.06	**0.005**		4	0.059	1.15	0.177		–	–	–	–		–	–	–	–	
Joint effect	4	0.345	2.45	**0.001**		4	0.044	1.13	0.103		–	–	–	–		–	–	–	–	
Microbes variables																				
Pure effect	1	0.013	1.12	0.383		–	–	–	–		1	− 0.026	0.72	0.682		1	− 0.022	0.77	0.711	
Joint effect	1	0.096	2.17	0.051		–	–	–	–		1	0.122	2.53	**0.011**		1	0.052	1.61	0.086	
Residual variation		0.618					0.871					0.838					0.958			

(a) Results of PERMANOVA tests, (b) environmental variables significantly correlated with community composition from NMDS ordinations, and (c) pure and joint effects of these environmental variables as derived from variation partitioning analyses. NMDS ordinations and variation partitioning are based on Bray–Curtis dissimilarity matrices constructed from OTU abundances. Values in bold are significant at *P* < 0.05

**Fig. 3:**
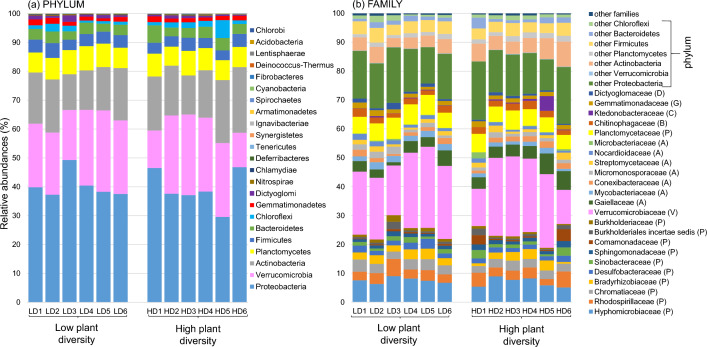
Bacterial community composition in low-diversity and high-diversity grassland plant communities. Relative abundances of bacteria, at **a** phylum and **b** family levels, in the soil are shown. The phylum corresponding to each family is indicated on the right in panel (**b**). Only the families with a relative abundance of > 2% for at least one of the 12 plots are shown

A closer look at bacterial community composition revealed no significant effect of plant community type on the bacterial phylum pattern (Fig. 3a). The same phyla were present in similar proportions in all plots regardless of the plant community type and its diversity level. Proteobacteria was the dominant phylum (40% on average), followed by Verrucomicrobia (23%) and Actinobacteria (17%). Similarities in the composition and proportion of families present can also be observed at the family level, regardless of the plant diversity level (Fig. 3b, Supplementary Table 4). Among the most abundant Proteobacteria families (i.e. families with a relative abundance of > 2% in at least one plot), the major families were Hyphomicrobiaceae (22% of the total most abundant Proteobacteria families), Rhodospirillaceae (11%), Chromatiaceae (10%) and Bradyrhizobiaceae (8%). The most abundant family of the Verrucomicrobia phylum was Verrucomicrobiaceae. Among the most abundant Actinobacteria families, Gaiellaceae dominated (31% of the total most abundant Actinobacteria families).

### Effects of plant community on fungal community diversity and composition

Fungal OTU richness, Chao1 and ACE diversity estimates were significantly higher in the high-diversity plots than in the low-diversity plots (Supplementary Table 3). Fungal community composition was significantly affected by plant community types (Table 2a). Fungal communities from low and high-diversity plots were differentiated on the first NMDS axis (Supplementary Fig. 4b). Both plant community and chemical variables were significantly correlated with fungal community composition, with CEC and dry shoot biomass having the strongest correlations (Table 2b). The variance in fungal community composition was significantly explained only by plant community in combination with chemistry (Table 2c, Fig. 2b). The variance in the community composition not explained by our variables reached 87%.

**Fig. 4:**
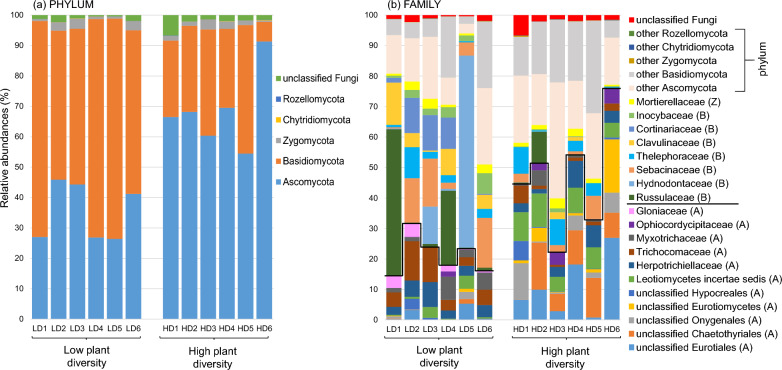
Fungal community composition in low-diversity and high-diversity grassland plant communities. Relative abundances of fungi, at **a** phylum and **b** family levels, in the soil are shown. Letters in parentheses indicate the phylum, including Ascomycota (A), Basidiomycota (B) and Zygomycota (Z). Only the families with a relative abundance of ≥ 3% for at least two of the 12 plots are represented. Glomeromycota were excluded from the datasets because they are presented independently in the paper

Fungal community composition at the phylum level showed significant differences between plant community types (Fig. 4a). Ascomycota and Basidiomycota were the predominant phyla, however, the relative abundance of Ascomycota was significantly higher in the high-diversity plots than in the low-diversity plots (t-test = 5.34, *P* value < 0.001; high-diversity = 69%, low-diversity = 35%), while the opposite effect was found for Basidiomycota (t-test = 5.12, *P* value < 0.001; high-diversity = 28%, low-diversity = 62%). At the family level, the most striking feature was the strong heterogeneity in the family composition depending on the plot considered (Fig. 4b, Supplementary Table 5). However, some significant effects of plant community type on family composition were evident. For example, the relative abundance of some families was higher in the high-diversity plots than in the low-diversity plots, e.g. Ophiocordycipitaceae, Leotiomycetes incertae sedis, and Eurotiales. Conversely, some families had lower relative abundances in high-diversity than in low-diversity plots, e.g. Trichocomaceae, Russulaceae, Sebacinaceae and Cortinariaceae.

**Fig. 5:**
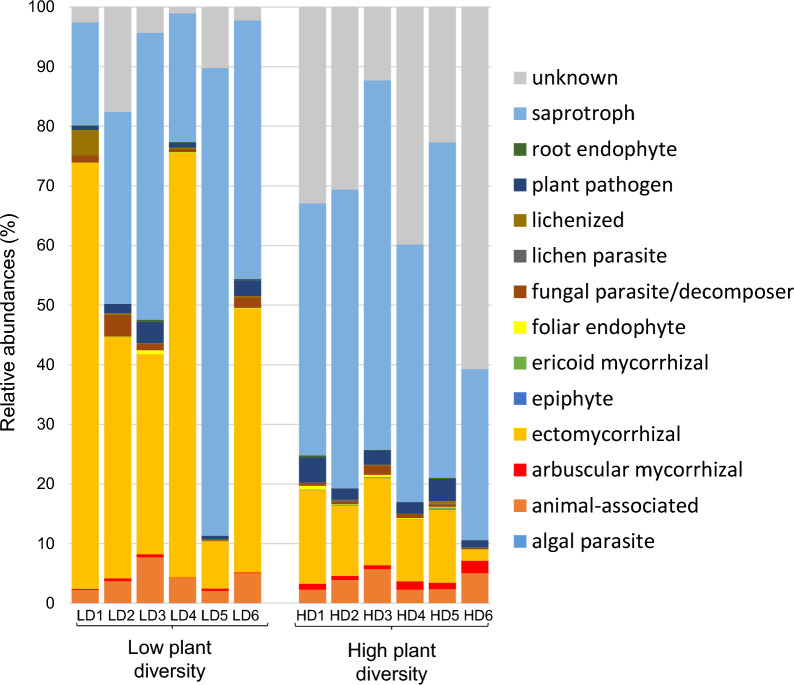
Relative abundances of ecological fungal groups in soils from low-diversity and high-diversity grassland plant communities

Among the fungal ecophysiological guilds identified, the plant community type had a significant effect on the relative abundances of the mycorrhizal guilds (Fig. 5). The most important effect was on ectomycorrhizal fungi (ECM), which were more abundant in the low-diversity plots than in the high-diversity plots (t-test = 3.35, *P* value = 0.018; high-diversity = 11%, low-diversity = 45%). In contrast, AMF were more abundant in the high-diversity plots (t-test = 3.74, *P* value = 0.009; high-diversity = 1.2%, low-diversity = 0.3%). However, we will not discuss this effect because the ITS2 general fungal primers are not reliable for quantifying AMF [58]. The effect of plant community type was not significant for all other identified guilds (0.087 < *P* values < 0.742).

### Effects of plant community on AMF community diversity and composition

AMF communities from soil and roots were independently analysed. OTU richness, Chao1 and ACE diversity estimates were significantly higher in the high-diversity plots than in the low-diversity plots for the soil AMF community only (Supplementary Table 3). Root AMF community diversity was not affected by plant community types (Supplementary Table 3). While the soil AMF community composition was clearly affected by the plant community type, the root AMF community was only slightly affected (*P* value = 0.046) (Table 2a, Supplementary Fig. 4c, d). Only few environmental variables were significantly correlated with the AMF community composition (Table 2b). Among plant community variables, plant richness was significantly correlated with both soil and root AMF community compositions, while dry shoot biomass was significantly correlated with soil AMF community composition. The last variable correlated with community composition was the AMF-to-fungi ratio for the soil AMF community and the total microbial biomass for the root AMF community. Surprisingly, none of the variables significantly explained the variance observed in the root AMF community composition, and the unexplained variance reached 96%. Conversely, the variance in the soil AMF community composition was significantly explained by both the effect of plant community combined with the joint effects of plant community and microbes, and the effect of microbes combined with the joint effects of microbes and plant community (Table 2c, Fig. 2c, d).

**Fig. 6:**
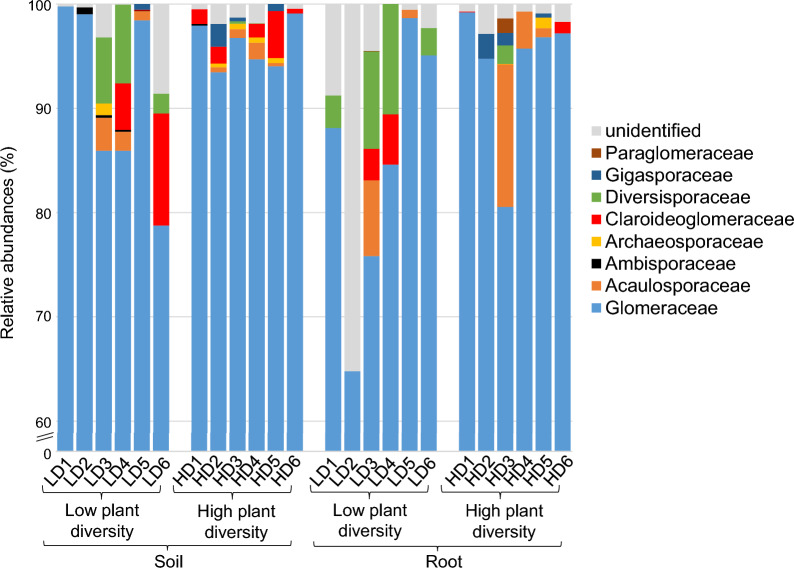
Relative abundances of AMF phyla in the soil and root compartments from the low-diversity and high-diversity grassland plant communities

Looking more closely at the AMF community composition, the Glomeraceae family largely dominated the communities both in soil and roots (soil: 94% of sequences; root: 89% of sequences) and at both plant diversity levels (Fig. 6). Claroideoglomeraceae and Diversisporaceae had lower relative abundances in high-diversity plots than in low-diversity plots, regardless of compartment (soil or roots), while the trend was reversed for Gigasporaceae, whatever the compartment.

## Discussion

Compared to previous studies located in managed grasslands, this work is unique in that it was conducted in an extensively managed semi-natural grassland (just mowing once per year) that possesses a European record of plant diversity. This grassland contains an extremely broad gradient in plant species richness in an area with homogeneous environment, which makes it an excellent model to study plant diversity effects on microbial communities. Furthermore, this study is unique in that it combines comprehensive description of the microbiome in terms of abundance and composition, i.e. composition and biomass of bacteria, general fungi and AMF fungi, with soil chemistry and composition of plant community and thus gives an opportunity to study drivers of multiple microbiome features within the experimental design.

The considerable local variability in plant species richness in the Čertoryje grassland is mainly driven by the presence of mature trees [86]. The low-diversity plots were closer to the trees than the high-diversity plots. As the pool of light-demanding plant species is much larger than the pool of shade-tolerant species in the study area [53], it is likely that higher levels of shading from nearby trees led to a decrease in plant diversity in the low-diversity plots by excluding light-demanding species [37]. Additionally, the lower shoot biomass in the low-diversity plots than in high-diversity plots could be due to competition for light, which reduces the aboveground plant biomass [75]. Nevertheless, Slachová [86] also found that soil moisture is lower in grasslands near trees than in treeless grassland patches. When we sampled the plots near trees, we found that their aboveground plant community was largely a subset of the species-rich grassland community in treeless areas, but we did not expect the belowground part to be so strongly dominated by tree roots. The dominant presence of the roots of trees, particularly *Quercus* and *Tilia*, which we detected using root sequencing in the low-diversity plots, could increase belowground competition. Competition could arise directly through the decrease in the pool of soil nutrients or water caused by tree roots and thus influencing the resources available to herbaceous species [78] or from the release of inhibitory root exudates by the shallow fine roots of trees, as previously demonstrated for *Quercus* sp. [12]. This could be the reason for the lower shoot and root water content that we measured in the low-diversity plots compared to the high-diversity plots, as well as for the lower soil moisture measured by Slachová [86] near trees. In addition, competition could be indirect through mycorrhizal symbionts. Indeed, plant genera interacting with ectomycorrhizal fungi (ECM) were dominant in the low-diversity plots, whereas plants interacting with arbuscular mycorrhizal fungi (AMF) dominated in the high-diversity plots. Such a competitive relationship between ECM and AMF could be explained by the depletion in resources by ECM to the detriment of AMF [5, 11]. The last potential level of indirect competition between trees and herbs could result from a change in soil chemistry caused by the trees. However, in our case, this is not the preferred hypothesis because we observed only small changes in soil chemistry according to the level of plant diversity and Slachová [86] did not find a consistent relationship between tree cover and pH in the wooded meadows of the White Carpathians.

The only associations between plant diversity and soil chemistry were higher contents of N and organic C in high-diversity than in low-diversity plots. The increase in soil organic C content along with the increase in plant species richness has already been demonstrated [3, 17, 82] and, in our study, could be due to the local increase in plant productivity of herbaceous species and microbial growth and activities. The higher soil fertility due to increased N content associated with the increase in plant diversity was observed in previous studies [21, 35]. In our study, the stimulation of microbial growth in the high-diversity plots was indicated by the difference in total microbial biomass, which could be attributed to higher bacterial biomass. This effect on bacterial biomass could result from higher contents of organic C and N in high-diversity than in low-diversity plots suggesting that plant communities release more nutrients that could also be more diverse due to the diversity of plant species [19]. This diversity of plant exudates could thus benefit to a broad range of bacteria. The fact that AMF biomass was five times higher in the high-diversity plot than in low-diversity plots likely resulted from the higher dependence of plant species in the high-diversity plots on AMF. The higher N content in the high-diversity plots could be due to the higher abundance of bacteria (particularly nitrogen fixing bacteria) and AMF, which are mainly involved in plant nitrogen acquisition [92]. This could also be due to the higher consumption of N by tree roots in the low-diversity plots. In turn, the high N content in the high-diversity plots could promote bacterial growth, as bacteria are largely dependent on this nutrient for growth. However, in contrast to a previous meta-analysis [19], the slightly higher total fungal biomass we observed in high-diversity plots was not significant, likely due to the increasing abundance of AMF competing with the rest of the fungal community [40, 67].

While bacterial biomass differed between the two grassland plant community types, there was no significant difference in soil bacterial composition and diversity between low-diversity and high-diversity plots. This finding confirms those previously found at the same locality by Navrátilová et al. [70]. Bacterial community composition was mainly driven by a combination of plant richness, soil chemistry and the abundance of AMF in the soil, although soil chemistry, particularly CEC and pH(H_2_O), explained most of the variance. This predominance of deterministic processes driving bacterial community composition in grasslands was already demonstrated by Guo et al. [41]. The strong effect of pH on bacterial community composition is also well known [49, 107]. As found by Navrátilová et al. [70], Proteobacteria and Actinobacteria are dominant in the Čertoryje grassland, but we also found Verrucomicrobia as a codominant phylum. In species-rich European grasslands, Proteobacteria and Actinobacteria were found to be the two phyla that mainly constitute the active bacterial community of the rhizosphere, while Verrucomicrobia were largely present in the soil but less involved in the active bacterial community [97]. Among the prominent Proteobacteria families, Hyphomicrobiaceae and Bradyrhizobiaceae belong to the Rhizobiales order, which is well known for its role in symbiotic nitrogen fixation [10], while Rhodospirillaceae and Chromatiaceae comprise more functionally diverse taxa [4, 48]. Finally, Gaiellaceae have been found to be ubiquitous and can adapt to a broad range of soil types [79], although little is known about their functions in soils [1].

In contrast to bacteria, the biomass of the fungal community did not differ between the two community types, while fungal diversity and composition did. Similarly to the data from the grassland biome by Yang et al. [106], we observed that the plots with high plant diversity also had higher soil fungal diversity. This lower soil fungal diversity in the plots with low plant diversity could partly result from the dominance of ECM in these plots. Both plant community and soil chemistry influenced fungal community composition. In particular, dry shoot biomass, which reflected the higher plant productivity in the high-diversity plots, and was found to drive fungal community composition, indicates the importance of the plant community in structuring the fungal community [16, 41]. Among the soil chemical variables affecting fungal community composition, CEC and pH(H_2_O) played an important role. These factors have previously been shown to be significant drivers in a forest biome [38, 62]. Both organic and inorganic C also played an important role in structuring the fungal community, as fungi are involved in the decomposition of soil organic matter [27]. Thus, among the environmental variables we selected, approximately 7% of the variance in fungal community composition was due to plant community and the joint effect of plant community and chemistry, and 87% of the variance remained unexplained. Among this unexplained variance, we can suppose that plant functional types (in particular trees versus herbs) played an important role [36]. Although our study included only a small fraction of environmental factors that could potentially affect fungal community composition, other studies on fungal community composition showed a high level of stochasticity in the fungal community assemblage [41, 59, 61]. As previously found in the Čertoryje grassland, Ascomycota and Basidiomycota were the dominant phyla [70], which is common in grassland ecosystems [16, 81]. The increase in Ascomycota and decrease in Basidiomycota in the high-diversity plots compared to the low-diversity plots was similar to the findings of nitrogen enrichment studies in grasslands [18, 56]. It is also consistent with the findings from experimental grasslands [85] highlighting that these differences in proportion of phyla result for the copiotroph and oligotroph characteristics of Ascomycetes and Basidiomycetes, respectively. Thus, in our study, this could be explained by the difference in N content that we observed. At lower taxonomic levels, it was difficult to observe consistent trends. Although some fungal families tended to increase or decrease depending on the plant diversity level, fungal composition appeared to be stochastic. The fungal ecophysiological guilds assigned to fungal genera confirmed what we found for the plant mycorrhizal types based on plant genera. Due to the predominance of tree roots in the low-diversity plots, they were richer in ECM than the high-diversity plots. In contrast to a previous study [24], we found no significant differences in the relative abundances of the other fungal guilds in relation to plant diversity levels.

Finally, we focused on AMF communities from soil and roots. Surprisingly, the AMF community differences between low-diversity and high-diversity plots depended on the compartment considered, i.e., soil or root. Abundance, diversity and composition of the soil AMF community were significantly affected by the grassland plant community types. The higher the plant diversity, the higher the diversity and abundance of the AMF community in the soil. In contrast, there was no difference between the two plant community types in root AMF community diversity. In addition, root AMF community composition was only slightly affected by plant diversity. This may indicate that plant diversity increases the abundance and diversity of AMF spores and mycelia in the soil, but not all of them colonize plant roots [44], probably due to competition with other AMFs established in the host plant [57], differential ability to colonize the host plant [43], or because they are not obligate symbionts [44]. In contrast to recent studies highlighting the importance of soil chemistry for AMF community composition [52, 93], in our study, AMF community composition in both soil and roots was not related to soil chemistry, likely because the study sites were selected to minimize the variation in soils. As expected, plant community played a role in the composition of both soil and root AMF communities, and significantly explained the variance in the soil AMF community composition, likely due to the higher proportion of AMF-dependent plant species in the high-diversity plots. However, it was interesting to see that none of our environmental factors significantly explained the variance in root AMF community composition and almost all of the variance (96%) remained unexplained. Indeed, it seems that other environmental factors might influence AMF community composition, such as temperature or precipitation [105] or plant shade tolerance [72]. Such factors can also be modulated by the presence of trees. However, it is also possible that more complex interactions between AMF and the rest of the microbial community, which was affected by the plant diversity, occurred and determined the AMF root community composition. Regardless of the compartment and the plant diversity level considered, the Glomeraceae family was largely dominant. This family was recognized as an indicator of undisturbed grasslands [68]. The Claroideoglomeraceae and Diversisporaceae families were found to increase in abundance in the shaded grassland sites compared to the non-shaded sites [72], which could explain why we observed a decrease in their relative abundance in the high-diversity plots not shaded by a tree. Finally, a higher abundance of Gigasporaceae in the high-diversity than in low-diversity plots could be because the niche optima for these taxa are at sites with high precipitation [28]. This could be the case in the high-diversity plots not shaded by trees and thus more exposed to precipitation than the low-diversity plots. Another explanation could be that Gigasporaceae are competitive mycorrhizal fungi capable of colonizing new hosts by themselves, and therefore might be favoured in the high-diversity plots where competition among AMFs in the soil is high [96, 98].

## Conclusions

We compared soil microbial communities between grassland plots with low plant diversity, located close to trees, and grassland plots with high plant diversity, located in nearby treeless areas. The biomass of bacteria was higher in plots with high plant diversity while bacterial diversity and composition did not differ between the two plant community types. Fungal community biomass did not differ between the two plant community types, but its composition differed and fungal diversity was higher in plots with high plant diversity. The response of AMF communities depended on their interaction with plants. AMF communities from soil had higher biomass and diversity in high-diversity plots, and their composition was affected by plant community types, whereas the diversity of root AMF communities was not affected by plant community types and their composition was only slightly affected. The factors influencing microbial community composition depended on the microbes considered. Bacterial community composition was mainly determined by soil chemistry, whereas fungal (including soil AMF) community composition was mainly determined by plant community characteristics but showed a high degree of stochasticity. Most of the unexplained variance in community composition involved root AMF communities. Further studies involving similar semi-natural, species-rich grasslands and analysing the functional role of microbial communities are needed for a better understanding of plant-soil-microbe interactions in grassland ecosystems.

### Supplementary Information

**Additional file 1.**

**Additional file 2.**

## Data Availability

The amplicon sequence data are available in the NCBI Sequence Read Archive under the accession number PRJNA891626.
