# Correction: Effect of plant communities on bacterial and fungal communities in a Central European grassland

**DOI:** 10.1186/s40793-024-00589-y

**Published:** 2024-07-31

**Authors:** Clémentine Lepinay, Tomáš Větrovský, Milan Chytrý, Pavel Dřevojan, Karel Fajmon, Tomáš Cajthaml, Petr Kohout, Petr Baldrian

**Affiliations:** 1 Institute of Microbiology of the Czech Academy of Sciences, Vídeňská, 1083, 14220 Prague 4, Czech Republic; 2 Department of Botany and Zoology, Faculty of Science, Masaryk University, Kotlářská 2, 61137 Brno, Czech Republic; 3 Regional Office Protected Landscape Area Bílé Karpaty, Nature Conservation Agency of the Czech Republic, Nádražní 318, 763 26, Luhačovice, Czech Republic

**Correction: Environmental Microbiome (2024) 19:42**

10.1186/s40793-024-00583-4

Following publication of the article, it came to the attention of the journal that the authors’ corrections to Table 2 had not been implemented during production of the article. As a result, there were incorrect values in the table. Specifically, in the third row from the bottom (‘Pure effect’ of ‘Microbe variables’), numbers in the row were incorrectly ordered and a minus sign had been added in error. The table has since been corrected in the published article. The publisher thanks you for reading this erratum and apologizes for any inconvenience caused.

