# Supplementary material for: Effect of plant communities on bacterial and fungal communities in a Central European grassland
Source: Environ Microbiome. 2024 Jun 20;19:42. doi: 10.1186/s40793-024-00583-4 (PMC11188233; doi:10.1186/s40793-024-00583-4)
Supplement: Supplementary file 1 — Additional file 1. [file 40793_2024_583_MOESM1_ESM.docx]

**Supplementary Data**

**Effect of plant communities on bacterial and fungal communities in a Central European grassland**

Clémentine Lepinay^1^, Tomáš Větrovský^1^, Milan Chytrý^2^, Pavel Dřevojan^2^, Karel Fajmon^3^, Tomáš Cajthaml^1^, Petr Kohout^1^, Petr Baldrian^1^

1. Institute of Microbiology of the Czech Academy of Sciences, Vídeňská 1083, 14220 Praha 4, Czech Republic

2. Department of Botany and Zoology, Faculty of Science, Masaryk University, Kotlářská 2, 61137 Brno, Czech Republic

3. Nature Conservation Agency of the Czech Republic, Regional Office Protected Landscape Area Bílé Karpaty, Nádražní 318, 763 26 Luhačovice, Czech Republic

Corresponding author: Clémentine Lepinay, [clementine.lepinay@gmail.com](mailto:clementine.lepinay@gmail.com)

**Supplementary Table 1**. Location and vegetation characteristics of the 12 studied plots in the Čertoryje grassland. LD and HD correspond to low and high levels of plant diversity, respectively. Total genus richness corresponds to the total number of genera per plot, taking into account the genera identified in the botanical survey of aboveground vegetation and adding the missing genera found by the sequencing analysis of plant roots.

**Supplementary Table 3**. Diversity indices for microbial communities in plots with low and high plant diversity in the Čertoryje grassland. Significant differences between low and high-diversity plots are indicated by a bold letter (P-value < 0.05).

**Supplementary Figure 1**. Example of a pair of plots in the Čertoryje grassland.

**Supplementary Figure 2**. Rarefaction curves of number of OTUs according to the sequencing depth for (a) plant communities, (b) bacterial communities, (c) general fungal communities, and (d) AMF communities from soil (solid lines) and roots (dashed lines). The vertical line indicates the rarefaction threshold.

**Supplementary Figure 3**. Relative abundances of putative mycorrhizal types based on the plant genera obtained from the sequencing of roots from each low and high plant diversity plots of the Čertoryje grassland. The putative mycorrhizal types were assigned based on Soudzilovskaia et al. (2020). NM: non-mycorrhizal, AM: arbuscular mycorrhizal, EcM: ectomycorrhizal.

**Supplementary Figure 4**. Two-dimensional non-metric multidimensional scaling (NMDS) representations of (a) bacterial, (b) fungal, (c) soil AMF and (d) root AMF communities in the low-diversity and high-diversity plots in the Čertoryje grassland. Vectors indicate the significant environmental variables.
